# Supplementary material for: On-chip Cherenkov radiation tuning in 3.2-14 THz
Source: Nat Commun. 2025 Aug 25;16:7921. doi: 10.1038/s41467-025-63368-4 (PMC12379205; doi:10.1038/s41467-025-63368-4)
Supplement: Supplementary file 1 — Supplementary Information [file 41467_2025_63368_MOESM1_ESM.pdf]

On-chip Cherenkov radiation tuning in 3.2-14 THz

Supplementary information

S1. Theoretical development of the graphene/hBN multilayer hyperbolic metamaterial (HMM) and CR properties in the HMM

To develop an HMM in the THz range, a kind of material that supports surface plasmons in the THz region and a kind of dielectric material with low transmission loss need to be selected[1]. Here, graphene is chosen as the plasmonic material due to its Fermi-level-controlled transverse magnetic (TM) surface plasmon properties[2], and hexagonal boron nitride (hBN) serves as the dielectric material considering its dielectric properties in the THz region[3].

The permittivity of graphene is theoretically obtained from Kubo theory[2]:

$$\sigma(\omega,\mu_c,\Gamma,T)=N_g\left(\sigma_{intra}+\sigma_{inter}\right) \tag{1}$$

where ω is the angular frequency, μ_c is the chemical potential of graphene, Γ is the scattering rate of graphene determined by the carrier mobility and Fermi velocity, T=298 K is the temperature, and N_g is the number of layers of graphene. σ_{intra} and σ_{inter} represent the conductivities contributed by the intraband and interband electron–phonon scattering processes, respectively. The permittivity and refractive index of graphene can then be obtained through[2]:

$$\varepsilon=1+\frac{i\sigma}{\omega\varepsilon_0 N_g t_g},\ n=\sqrt{\varepsilon} \tag{2}$$

where t_g=0.334 nm is the thickness of a single layer of graphene.

Additionally, the permittivity of hBN is obtained from the Lorentz oscillator model[3]:

$$\varepsilon_u=\varepsilon_{\infty,u}\left[1+\frac{\omega_{LO,u}^2-\omega_{TO,u}^2}{\omega_{TO,u}^2-\omega\left(\omega+i\gamma_m\right)}\right] \tag{3}$$

where the subscript u denotes two directions orthogonal ($\perp$) or parallel ($//$) to the hBN sliding plane. The parameters have values of $\varepsilon_{\infty,//}$=4.87, $\varepsilon_{\infty,\perp}$=2.95, $\omega_{LO,//}$=1610 cm^{-1}, $\omega_{TO,//}$=1367 cm^{-1}, $\omega_{LO,\perp}$=828 cm^{-1}, $\omega_{TO,\perp}$=783 cm^{-1}, $\gamma_\perp$= 4 cm^{-1} and $\gamma_{//}$=5 cm^{-1} [3].

The graphene-hBN multilayer structure can be considered an anisotropic material with ε_x, ε_y and ε_z representing the permittivity components in the x, y and z directions,

respectively. Based on the permittivity of graphene and hBN, the anisotropic permittivity of the HMM can be obtained through the Maxwell–Garnett formula[4]:

$$\begin{cases} \varepsilon_x = \dfrac{\varepsilon_m \varepsilon_d}{(1-p)\varepsilon_m + p\varepsilon_d} \\ \varepsilon_y = \varepsilon_z = p\varepsilon_m + (1-p)\varepsilon_d \end{cases} \tag{4}$$

Supplementary Fig. 1 shows the calculated permittivity distribution of the multilayer structure in the THz region, and the blue shaded area (f = 1-18.8 THz) represents the type-II hyperbolic region where $\varepsilon_x>0$ and $\varepsilon_y=\varepsilon_z<0$.

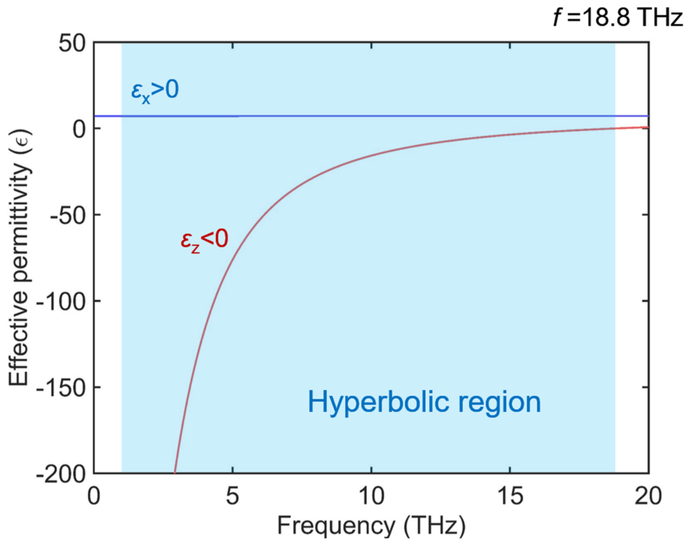

Supplementary Fig. 1 Calculated anisotropic permittivity dispersion of the graphene/hBN multilayer structure. The thicknesses of graphene and hBN are set to 3 nm and 45 nm, respectively. In the blue shaded region of 1-18.8 THz, the multilayer structure can be treated as a type-II HMM with $\varepsilon_x>0$ and $\varepsilon_y=\varepsilon_z<0$.

Our previous work indicated that thresholdless CR can be generated in the HMM when the velocity of free electrons satisfies[5]:

$$u_0 < \frac{c}{\sqrt{\varepsilon_x}} \tag{5}$$

where u_0 is the velocity of electrons and c is the light velocity in vacuum. The calculated maximum velocity is approximately 1.12×10^8 m/s (namely, approximately 40 keV in kinetic energy) in the hyperbolic region, so free electrons with an energy of 1.4-2.6 keV can excite THz CR in the HMM.

Furthermore, the ability of the HMM to compress the EM modes can be characterized by the effective refractivity n_{eff}[5], which can reach 10-70 in the hyperbolic region when 1.4 keV<E<2.6 keV. Such a large n_{eff} represents a strong wavelength compression capability, which helps integrate the THz source into a chip hundreds of

micrometers in size.

By the way, to homogenize the multilayer HMM made of alternating graphene and hBN, the thickness of each layer need to be much smaller than the effective wavelength λ_{eff} of excited CR, which can be calculated by [5]:

$$\lambda_{eff} = \frac{\lambda_0}{\sqrt{\varepsilon_z + \left(1 - \frac{\varepsilon_z}{\varepsilon_x}\right)\left(\frac{c}{u_0}\right)^2}} \tag{6}$$

where λ_0 is vacuum wavelength of the THz wave, ε_x and ε_z are the permittivity component of the HMM in the *x*- and *z*-directions, u_0 is the velocity of the electrons and *c* is the light velocity in vacuum. Here, the thickness of graphene and hBN are selected as 1-5 nm and 40-45 nm to satisfy the homogenization condition.

S2. Theoretical analysis of the wavevector matching

Considering the anisotropic permittivity of the HMM, the dispersion relation of the CR in the HMM can be written as[6]:

$$\frac{k_x^2}{\varepsilon_{z(y)}} + \frac{k_{z(y)}^2}{\varepsilon_x} = \left(\frac{\omega}{c}\right)^2 \tag{7}$$

where $\mathbf{k_x}$, $\mathbf{k_y}$ and $\mathbf{k_z}$ represent the CR wavevectors in x, y and z directions, ω is the angular frequency. Although CR modes have wavevector components in three directions, only $\mathbf{k_x}$ and $\mathbf{k_z}$ are considered in the following analysis because $\mathbf{k_y}$ makes no contribution to the radiation extracted into free space through wavevector compensation by the grating, which is uniform in the *y*-direction. The 3D dispersion surface of k_x-k_z-ω is drawn as the red surface in Fig. 1b, and only the $|\mathbf{k_z}|>0$ region is considered for free electrons traveling along the $+z$ direction. The dispersion line of the evanescent field surrounding a free electron in *z*-direction (namely, $\omega=u_0\cdot|\mathbf{k_z}|$) is depicted as the black line.

For a certain frequency $\omega=\omega_0$, the k_x-k_z section view of the 3D red surface in Fig. 1b is a hyperbolic curve, which is shown as the red line in Fig. 1b and represents the isofrequency contour of the HMM. The minimum $|\mathbf{k_z}|=\varepsilon_x^{1/2}\cdot|\mathbf{k_0}|$, marked by the red hollow circle, corresponds to the maximum velocity for generating CR as stated in Section S1. In supplementary Fig. 2a, the black dashed line denotes the value of the wavevector $\mathbf{k_z}$ of the evanescent field surrounding the free electron and the longitudinal wavevector of the CR in the HMM at frequency $\omega=\omega_0$, and the wavevectors $\mathbf{k_z}$ and $\mathbf{k_{CR}}$ are depicted as violet and blue arrows, respectively.

Considering that the grating period lies in the z-direction, the process of wavevector compensation for free-space radiation depends only on $\mathbf{k_z}$. Thus, supplementary Fig. 2b illustrates a section view of the ω-k_z plane (namely, the k_x=0 plane) in Fig. 1b, which depicts the wavevector matching between the evanescent field of the free electron, CR in the HMM and free-space radiation. The red dashed-dotted line (same as that in Fig. 1b) represents where the end point of the $\mathbf{k_z}$ of the CR in the HMM could be when $\omega=\omega_0$. The dashed and solid lines represent the dispersion of the free-space light wave ($\omega=c\cdot|\mathbf{k_0}|$) and the evanescent field surrounding the free electron ($\omega=u_0\cdot|\mathbf{k_z}|$), respectively. The red dot is the intersection point between the red dashed-dotted line and the black solid line in supplementary Fig. 2b, which represents the end point of the wavevector $\mathbf{k_z}$ of the excited CR in the HMM. Furthermore, using the grating, the longitudinal wavevector $\mathbf{k_z}$ of the CR in the HMM can be completely compensated by $2\pi n/p$ (shown as the red dashed arrow in Fig. 1b and supplementary Fig. 2b), and THz radiation propagating along the -x direction is extracted into free space, as stated in Fig. 2 of the main text.

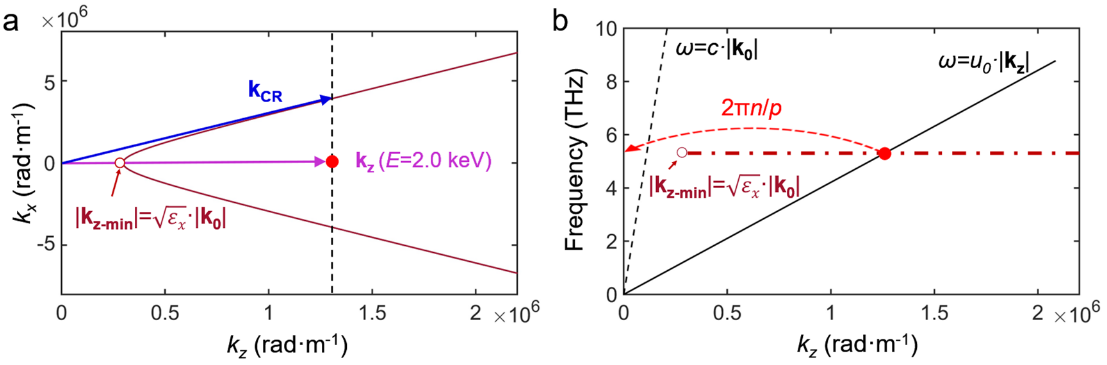

Supplementary Fig. 2 Theoretical analysis of the wavevector compensation. a 2D sketch of the wavevector matching for the evanescent field surrounding a free electron and CR in the HMM when $\omega=\omega_0$. The red hyperbolic curve represents the isofrequency contour of the mode in the HMM when f_0=5.61 THz, which has the minimum $|\mathbf{k_{z\text{-}min}}|=\varepsilon_x^{1/2}\cdot|\mathbf{k_0}|$ (denoted by the red hollow circle), with $|\mathbf{k_0}|=\omega/c$. The violet arrow $\mathbf{k_z}$ is the wavevector of the evanescent field surrounding free electrons when E=2.0 keV. The wavevector of the CR in the HMM $\mathbf{k_{CR}}$ due to the wavevector matching is shown as the blue arrow. The red hyperbolic curve, red circle and blue arrow are also drawn in Fig. 1b in the main text. **b** Section view of the k_x=0 plane in Fig. 1b depicting the wavevector matching. The red dashed-dotted line corresponding to that in Fig. 1b is the possible end point of the $\mathbf{k_z}$ of the CR in the HMM when $f=f_0$, which is also discussed in Fig. 2b. The dashed and solid black lines represent the light line ($\omega=c\cdot|\mathbf{k_0}|$) and dispersion line of the evanescent field surrounding the free electron ($\omega=u_0\cdot|\mathbf{k_z}|$), respectively. The red circle corresponds to that in **a**, and the red solid dot represents the end point of the $\mathbf{k_z}$ of the excited CR in the HMM. The red dashed arrow represents the wavevector compensation by the grating of $2\pi n/p$; thus, the CR in the HMM can be extracted into free space and propagate along the -x direction.

S3. Design of planar electrodes and characteristic of the vacuum current

In terms of material selection, molybdenum (Mo) has a high melting point up to ~2615 °C and a low electron work function down to 4.4 eV. In addition, the fabrication of Mo structures is relatively easy, so Mo has been widely used for free-electron emitting structures, such as Spindt cathodes[7]. Thus, we select Mo as the material for the planar electrodes of the radiation chip.

The cathode-anode structure is designed for free-electron beam generation in the THz radiation chip. As shown in Fig. 1a, the width of the cathode in the *y*-direction is set to 50 μm so that it can generate a relatively large-area belt-like beam with a current reaching tens of microamperes. Furthermore, to decrease the high voltage threshold for electron emission, a zigzag structure is adopted for the front of cathode. A row of tips with a curvature radius of 2 μm is fabricated through UV lithography for convenience of electron emission, as shown in supplementary Fig. 3.

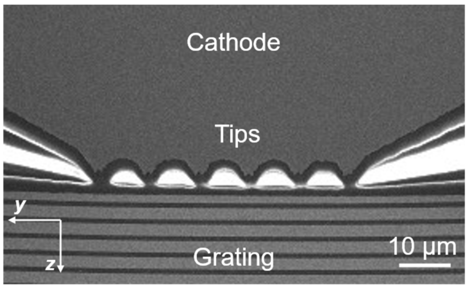

Supplementary Fig. 3 SEM image of the zigzag cathode. The planar cathode has a thickness of 700 nm (350 nm SiO_2 and 350 nm Mo) in the *x*-direction and a width of 50 μm in the *y*-direction. The front of the cathode is made of a row of tips with a curvature radius of 2 μm fabricated by UV lithography

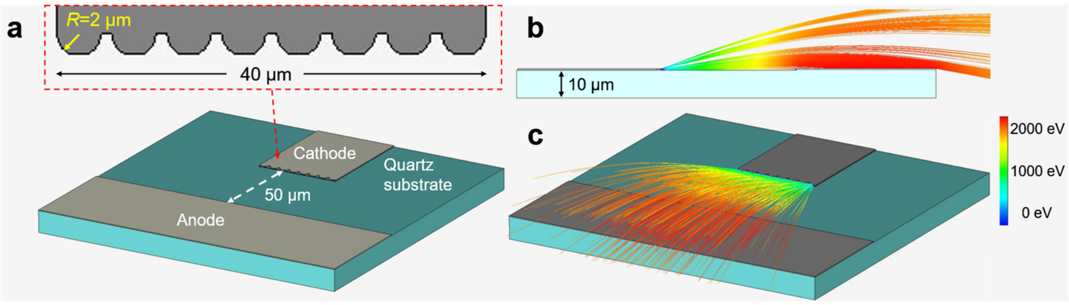

Supplementary Fig. 4 Simulated free electron trajectories a The simulation model of the planar cathode and anode. The width of the cathode is about 40 μm, and the-zigzag structure in the front of the cathode has a curvature radius of *R*=2 μm in each tip. The substrate is a 10μm-thick quartz. **b**, **c** The simulated free electron trajectory distribution with the method of Fowler-Nordheim formula and particle track. A belt-like free electron beam is shown between cathode and anode.

The field emission characteristic is calculated and simulated. Field emission vacuum current can be modeled by Fowler-Nordheim formula[8], based on which the particle trajectory can be simulated with the method of particle track. Supplementary

Fig. 4a is the structure model of the planar cathode and anodes which is consistent with the fabricated samples. Supplementary Fig. 4b and c illustrate the side and perspective view of the trajectory of free electrons with DC bias *V*=2 kV, respectively. It is shown that the vacuum current has a belt-like shape between the cathode and anode, which can help to improve the efficiency of the interaction between free electrons and HMM.

In experiment, the performance of the free electron emission is characterized by the I-V curve which shows the vacuum current as a function of the voltage in supplementary Fig. 5. The cross points are the measured vacuum current while the red line is a third-order polynomial fitted line, which shows the general characteristic of the free-electron-emission of the electrodes.

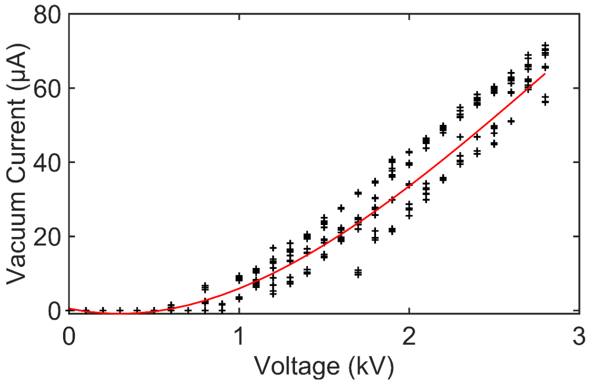

Supplementary Fig. 5 Measured vacuum current as a function of voltage applied on electrodes. The cross points represent the measured current values of three samples (perform three repetitions of the measurement for each sample), which are fitted through a third-order polynomial as illustrated by the red line.

S4. Measurement of the radiation power

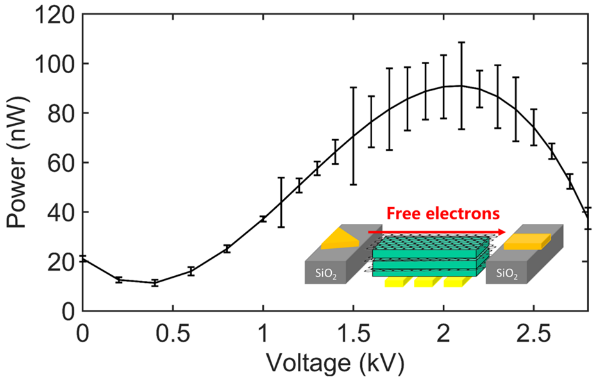

Supplementary Fig. 6 Measured power of the chips with graphene/hBN HMM. The black line is obtained by the third-order polynomial fitting of the measured power of three different chips The error bars represent the mean squared error of the fitted and measured values, length of error bar $l = (1/n) * \sum_{i=1}^{n}(p_i - \bar{p})^2$, where n is the total experimental runs, p_i is the measured power for i^{th} measurement and $\bar{p}$ is the fitted power value.

In experiment, the measured maximum radiation power of different chips as a function of the voltage (electron energy) is depicted in supplementary Fig. 6. The error bar shows the mean squared error of the measured power values of three different chips and the black line is obtained by the third-order polynomial fitting. According to the measured results, the maximum radiation power can reach 80-100 nW when voltage lies in 1.8-2.2 kV.

In theory, the radiation power should have shown apparent positive correlation with the vacuum current. However, by comparing supplementary Fig. 5 and supplementary Fig. 6, there exists positive correlation only when V <2 kV, while an abnormal decrease occurred when V >2 kV. The most possible reason is that relatively high voltage (which means high energy of free electrons) will cause a rapid heat accumulation and structural damage on the HMM, leading to a reduction of the CR intensity. To avoid this issue, further experiment could adopt discontinuous voltage bias (for example: rectangular-wave voltage) instead of DC voltage applied on the electrodes, which can help to reduce the heat generation and accumulation.

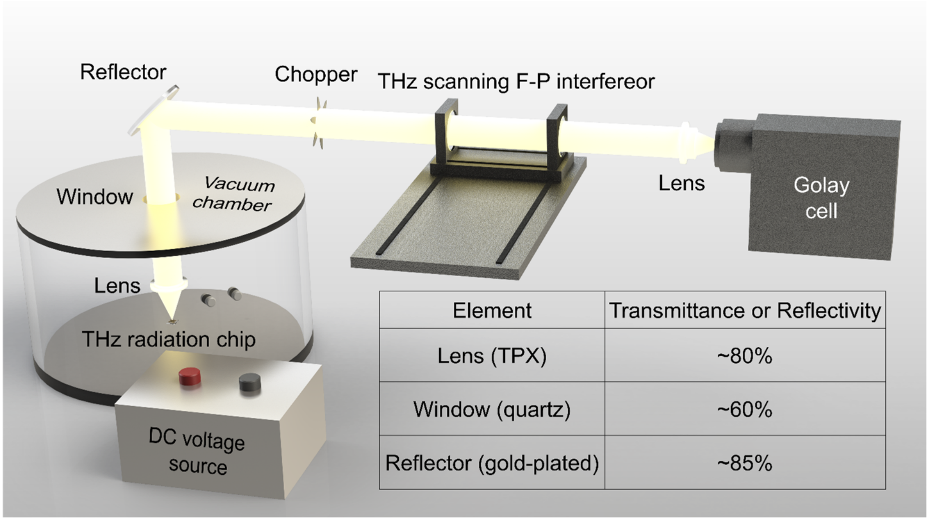

Supplementary Fig. 7 Sketch of the experimental measurement system and estimated loss for each element.

To obtain the original radiation power of the chip, the loss of the optical path is estimated. As shown in supplementary Fig. 7, considering the transmittance or reflectivity of the two TPX lenses (~80% for each), one gold-plated reflective mirror (~85%), one quartz window (~60%) and 80 cm-long propagation length (ignored), only ~25-30% of the power emitted by the chip can be collected by the Golay cell. As a result, the experimentally emitted THz radiation can reach 370-445 nW. To enhance the radiation power, we could increase the free electron current interacting with HMM and

apply more efficient extraction structures. The measured results are highly consistent with the calculated results, with an error in the allowable range. The difference between the measured and simulated powers may originate from insufficient electron acceleration, electron beam diffusion and fabrication errors.

S5. Corresponding grating period *p* and free-electron kinetic energy *E* of the numbered samples in Fig. 2a

p (μm)	*E* (keV)	Sample Number
6	1.4	1
6	2.0	2
6	2.6	4
5	1.4	3
5	2.0	5
5	2.6	7
4	1.4	6
4	2.0	9
4	2.6	10
3	1.4	8
3	2.0	11
3	2.6	13
2	1.4	12
2	2.0	14
2	2.2	15

S6. Analysis of the radiation power

(1) Power calculation by Frank-Tamm formular

The CR power density could be theoretically calculated through the Frank–Tamm formula [9-10]:

$$dW = \frac{q^2 l}{4\pi}\mu(\omega)\omega\left(1-\frac{c^2}{u_0^2 n^2(\omega)}\right)d\omega \tag{8}$$

where W is the total energy of the excited CR, q is the charge of the charged particle (bunch), l is the interaction length between the charged particle and the HMM, $\mu(\omega)$ represents the magnetic conductivity, and $n(\omega)$ represents the refractive index

distribution. The calculated CR power density is at the level of 10^5 W/m^2 according to the above equation.

To compare the radiation power in theory and experiment, the THz radiation power P coupled into free space can be obtained through the following formula:

$$P = \rho \cdot S \cdot \eta \tag{9}$$

where ρ is the power density of the CR generated by free electrons in the HMM, S is the effective coverage area of the HMM on the grating, and η is the extraction efficiency of the gold grating. According to the theoretical calculations, the maximum ρ can reach more than 10^5 W/m^2, and η is approximately 0.6%. Fig. 1c illustrates that S is approximately 30 μm× 30 μm=900 μm^2. Thus, the power of the radiation extracted into free space is estimated to be approximately P=540 nW.

(2) Power calculation by ordinary and extraordinary wave analysis

Recently, a new method for CR power calculation in anisotropic materials is raised through dividing the radiation into ordinary and extraordinary waves[11]. According to this method, we set the distance between the electron trajectory and the top surface of the HMM to be 200 nm, E=2 keV, vacuum current I=30 μA and interaction distance of 30 μm. The calculated radiation power is about 537 nW which is similar to the result from F-T formula.

(3) Power simulation by FDTD

Moreover, the power density is simulated for comparison by setting two kinds of electron shapes. A single bunch of electron with charge of 1.6×10^{-17} C, FWHM=130 fs and full width of 200 fs excites the pulsed radiation with maximum power density of 10^5 W·m^{-2}. For the DC vacuum current of I=80 μA, the excited power density of CR is also 10^4-10^5 W·m^{-2}. Both the simulation results show consistency with the above calculated and experimentally measured ones.

S7. Experiment and analysis of the radiation mechanism

To reveal the origin of the radiation, controlled experiments are conducted to indicate that the graphene/hBN HMM plays an important role on THz generation. With the planar electrodes and grating structure stay consistent with the experimental group in supplementary Fig. 6, the chip in the first control group shown in supplementary Fig. 8a has no graphene or hBN layer transferred, and the measured power is actually the noise in the level of 10-25 nW without any obvious change when varying voltage loaded on the electrodes. Considering Smith-Purcell radiation (SPR) might be excited based

on the structure in this condition[12-15], the control experiment indicates that the measured radiation in supplementary Fig. 6 is not originated from SPR. The reason that SPR cannot be observed is that, without HMM and its high density of state (DOS)[5], the scattering efficiency of the evanescent field surrounding electrons by the grating will decrease a lot, leading to an undetectable SPR in the measurement system.

Similarly, supplementary Fig. 8b shows the structure diagram and the measured power of chips in the second control group with only one layer of graphene (thickness of 1-5 nm) transferred, while the planar electrodes and grating structure stay consistent with the experimental group. It is shown that there is also no obvious radiation detected. This condition corresponds to the graphene-plasmon-related radiation which could be excited by free electrons, such as radiation from the THz plasmon[16], hot-carrier-induced plasmon[17] or others. As a result, by comparing the measured results between the experimental and control group, the mechanism of THz CR in HMM can be validly proved in experiment.

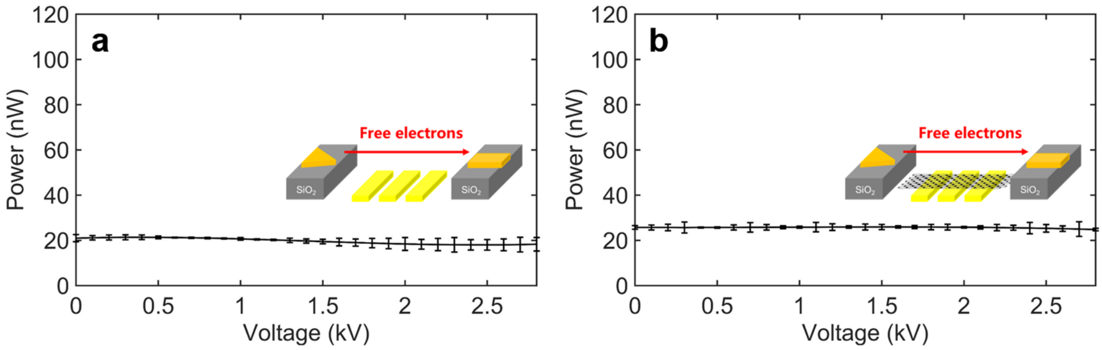

Supplementary Fig. 8 Measured radiation power of the control groups. a Measured power of the chips without graphene/hBN transferred in the first control group. **b** Measured power of the chips with only one layer of graphene (thickness of 1-5 nm) transferred in the second control group. The planar electrodes and grating structure for the chips in **a**, **b** and supplementary Fig. 6 are the same. The black line is obtained by the third-order polynomial fitting of the measured power of three chips. The error bars represent the mean squared error of the fitted and measured values, length of error bar $l = (1/n) * \sum_{i=1}^{n}(p_i - \bar{p})^2$, where n is the total experimental runs, p_i is the measured power for i^{th} measurement and $\bar{p}$ is the fitted power value.

S8. Analysis of the linewidth

Considering that the core area of the chip (~100 μm×100 μm) is comparable to the wavelength of THz wave (~20-100 μm), the chip can be approximated as a point light source emitting THz waves with different frequency components for various polar angles. Therefore, the width of the collected THz wave depends on the NA of the collecting system. In Fig. 3b and c, the extracted spectrum is simulated by setting a frequency probe under the device with distance of 100 μm. While, in experiment, the

collection is conducted through a THz lens with diameter of 1 inch and focal length of 10 cm as shown schematically by supplementary Fig. 7. Therefore, the linewidth of the measured results (~40 GHz, see Fig. 2d and e) is much narrower than that of the simulated one (Fig. 3b and c) because of the smaller effective NA in experiment.

S9. Design of the arrayed chip

To expand the tunable range or enhance the radiation power, the arrayed structure is adopted and fabricated. Supplementary Fig. 9a shows the chip fixed onto a copper holder to avoid sliding. The switchable cathode and anode probes are set to supply voltage bias and choose the radiation unit. The inset of supplementary Fig. 9a shows a partially enlarged figure of four radiation units, among which the working one is selected by switching the cathode probe. The arrayed structure can be fabricated at the same time and need no cutting and splicing operations. Supplementary Fig. 9b and c are the detailed images of one unit without and with HMM fabricated.

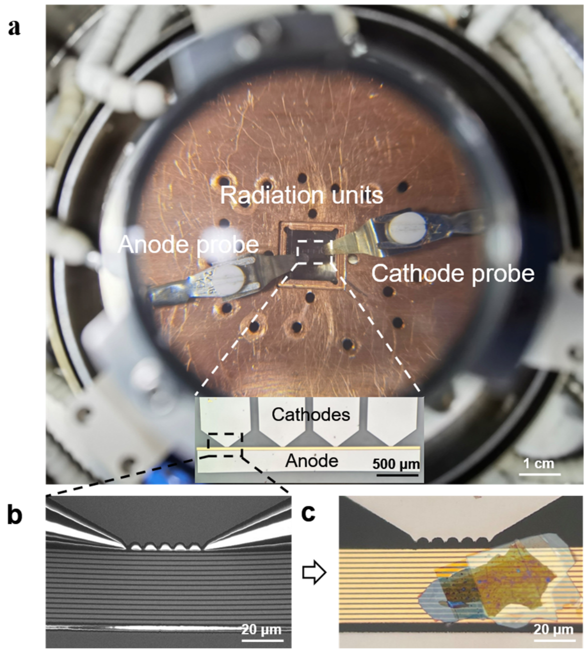

Supplementary Fig. 9 Image of the arrayed chip on the sample holder. a The chip is fixed onto a copper holder and the cathodes and anode are connected to probes which can supply voltage bias and switch to different radiation units. The inset is a partial enlargement of the chip, which shows four radiation units with switchable cathodes. **b** SEM image of the core area of one radiation unit, which contains cathode, anode and gold grating. After the HMM is prepared and transferred, the image of the radiation unit is shown in **c**.

S10. Particle-in-cell finite difference time domain (PIC-FDTD) simulation

Numerical simulation is conducted with the particle-in-cell finite difference time domain method. The graphene and hBN are modeled as 2 nm×10 μm×40 μm (for

graphene) and 43 nm×10 μm×40 μm (for hBN) flakes in the *x*-*y*-*z* directions. The permittivities of graphene and hBN are obtained from the Kubo formula[2] and Lorentz oscillator model[3], respectively, as stated in Section S1. For convenience in observing the CR field distribution, 30 layers of graphene and 30 layers of hBN are alternatively stacked to form the HMM.

In the simulation, CR is excited by electron bunches with a charge of 1.6×10^{-17} C and FWHM of 130 fs (full width of 200 fs) corresponding to a mean effective current of 80 μA, whose trajectory lies 100 nm above the top surface of the HMM along the *z*-axis, and the kinetic energy of the bunch is controlled from 1.4 to 2.6 keV.

The gold grating cleaving to the bottom surface of the HMM has a thickness of 60 nm, a slit width of 1 μm, and a period varying from 2 μm to 6 μm depending on the expected radiation frequency. A probe is set at the center of the HMM to detect the intensity and spectrum of CR in the HMM, while another probe is set 100 μm below the grating to detect the intensity and spectrum of the THz radiation extracted into free space.

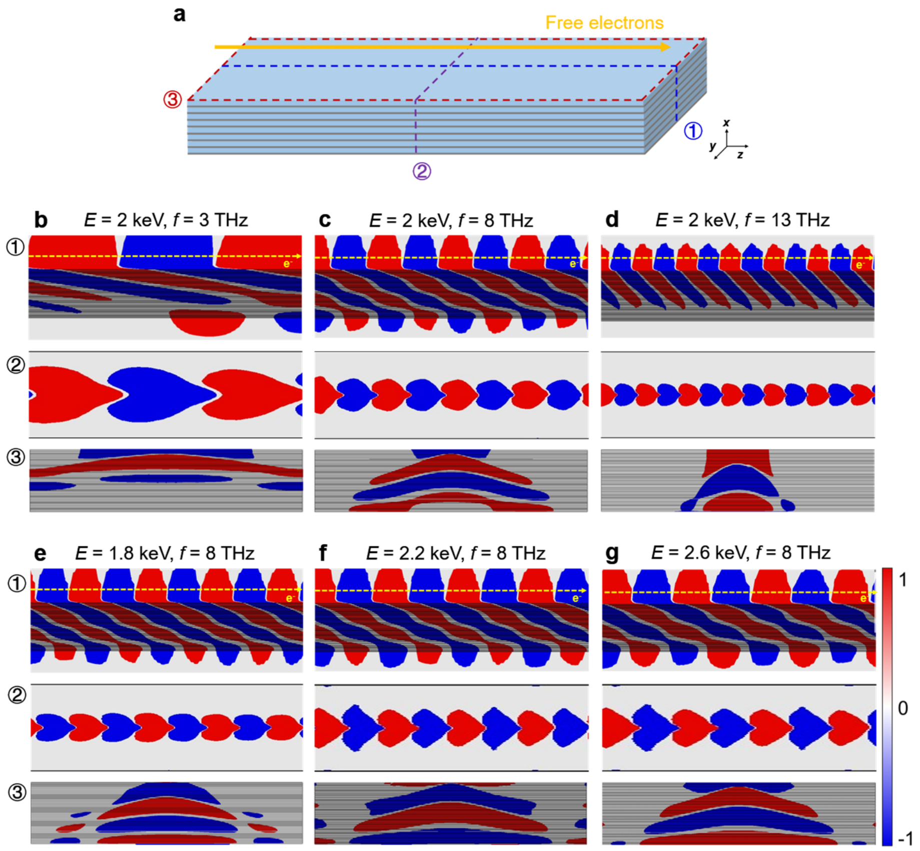

Supplementary Fig. 10 Simulated field distribution in different sections. a Sketch of the simulation model and the sections selected to observe the field distribution. Section ①, ② and ③ corresponds to the *x*-*z*, *x*-*y* and *y*-*z* sections, respectively. Simulated CR field contours (normalized $|E_z|$ components) in the HMM are depicted with **b** E=2 keV, f=3 THz. **c** E=2 keV, f=8 THz. **d** E=2 keV, f=13 THz. **e** E=1.8 keV, f=8 THz. **f** E=2.2 keV, f=8 THz. **g** E=2.6 keV, f=8 THz.

The simulated field distribution is depicted in supplementary Fig. 10. supplementary Fig. 10a shows the sketch of the simulation model and the observation sections, and supplementary Fig. 10b-g show the field contours of CR in HMM with various E and f. It is obvious that for a fixed E, CR wavelength decreases with higher f, and for a fixed f, CR wavelength increases with larger E, which further shows the tunability of the THz radiation extracted by the grating.

S11. Analysis of the redshift phenomenon of CR spectrum

In this study, the experimentally measured results show redshift deviation from the numerical and theoretical ones. The insufficient acceleration of the free electrons might be the major reason of the red-shift of the measured radiation frequency. Here we can give some possible methods to reduce the red-shift. Firstly, the free electron emitter with grid electrode could be applied, providing a DC bias between cathode and grid which can preliminarily accelerate electrons and improve the acceleration capability. Secondly, if the electron emitter is not integrated onto the chip, the free electron beam with better monochromaticity, such as that in SEM, could be selected for satisfying the theoretical analysis best.

S12. Analysis of the tunability

An on-chip CR emitter was first reported for the wavelength range of 500-900 nm[5]. In that paper, the radiation wavelength seemed to be determined only by the grating period, and the electron velocity had little influence. Nevertheless, we can tune the wavelength over a relatively large range here. This can be understood and compared based on the wavevector matching condition.

According to the wavevector compensation relation $\omega/u_0=2\pi n/p$, when the electron kinetic energy is tuned from 0.46 keV to 1.4 keV, the central frequency of the radiation in the previous work is estimated to be shifted by ~17.7 THz (~40 nm around $\lambda_0\approx800$ nm)[5]. Considering that the radiation spectrum was Gaussian fitted with the data obtained from a spectrometer, the ~40 nm wavelength shift was not obvious compared with the relatively broad output spectrum, as shown in Fig. 2b of that paper[5]. However, in this work, the measured tunable range spans 3.2-14 THz, while the linewidth is only approximately 40 GHz, so an apparent frequency shift with electron energy can be observed.

Reference

[1] Korzeb, K., Gajc, M. & Pawlak, D. A. Compendium of natural hyperbolic materials. *Opt. Express* **23**, 25406-25424 (2015).

[2] Luo, X., Qiu, T., Lu, W. & Ni, Z. Plasmons in graphene: Recent progress and applications. *Mater. Sci. Eng. R Rep.* **74**, 351-376 (2013).

[3] Li, N. *et al*. Direct observation of highly confined phonon polaritons in suspended monolayer hexagonal boron nitride. *Nat. Mater.* **20**, 43–48 (2021).

[4] Starkov, I. A. & Starkov, A. S. Maxwell–Garnett model for thermoelectric materials. *Int. J. Solids Struct.* **202**, 226-233 (2020).

[5] Liu, F. *et al*. Integrated Cherenkov radiation emitter eliminating the electron velocity threshold. *Nat. Photonics* **11**, 289–292 (2017).

[6] Poddubny, A., Iorsh, I., Belov, P. & Kivshar, Y. Hyperbolic metamaterials. *Nat. Photonics* **7**, 948-957 (2013).

[7] Spindt, C. A. A Thin-Film Field-Emission Cathode. *J. Appl. Phys.* **39**, 3504–3505 (1968).

[8] Jensen, K. L. *et al*. Electron emission from a single spindt-type field emitter: Comparison of theory with experiment. *Appl. Surf. Sci* **111**, 204-212 (1997).

[9] Frank, I. & Tamm, I. E. Coherent Visible Radiation of Fast Electrons Passing Through Matter. *CR Acad. Sci. URSS* **14**, 109-114 (1937).

[10] Tamm, I. E. General Characteristics of Vavilov-Cherenkov Radiation. *Science* **131**, 206-210 (1960).

[11] Adiv, Y. *et al*. Observation of 2D Cherenkov Radiation. *Phys. Rev. X* **13**, 011002 (2023).

[12] Mizuno, K. *et al*. Experimental evidence of the inverse Smith–Purcell effect. *Nature* **328**, 45–47 (1987).

[13] Kaminer, I. *et al*. Spectrally and spatially resolved Smith-Purcell radiation in plasmonic crystals with short-range disorder. *Phys. Rev. X* **7**, 011003 (2017).

[14] Ye, Y. *et al*. Deep-ultraviolet Smith-Purcell radiation. *Optica* **6**, 592-597 (2019).

[15] Yang, Y. *et al*. Photonic flatband resonances for free-electron radiation. *Nature* **613**, 42–47 (2023).

[16] Zhan, T. *et al*. Tunable terahertz radiation from graphene induced by moving electrons. *Phys. Rev. B* **89**, 245434 (2014).

[17] Kaminer, I. *et al*. Efficient plasmonic emission by the quantum Čerenkov effect from hot carriers in graphene. *Nat. Commun.* **7**, 11880 (2016).
